# Joint model robustness compared with the time-varying covariate Cox model to evaluate the association between a longitudinal marker and a time-to-event endpoint

**DOI:** 10.1186/s12874-019-0873-y

**Published:** 2019-12-03

**Authors:** Maeregu W. Arisido, Laura Antolini, Davide P. Bernasconi, Maria G. Valsecchi, Paola Rebora

**Affiliations:** Center of Biostatistics for Clinical Epidemiology, School of Medicine and Surgery, University of Milano-Bicocca, Via Cadore 48, Monza, 20052 Italy

**Keywords:** Cox model, Joint model simulation, Longitudinal biomarker, Random effects model, Time-varying covariate

## Abstract

**Background:**

The recent progress in medical research generates an increasing interest in the use of longitudinal biomarkers for characterizing the occurrence of an outcome. The present work is motivated by a study, where the objective was to explore the potential of the long pentraxin 3 (PTX3) as a prognostic marker of Acute Graft-*versus*-Host Disease (GvHD) after haematopoietic stem cell transplantation. Time-varying covariate Cox model was commonly used, despite its limiting assumptions that marker values are constant in time and measured without error. A joint model has been developed as a viable alternative; however, the approach is computationally intensive and requires additional strong assumptions, in which the impacts of their misspecification were not sufficiently studied.

**Methods:**

We conduct an extensive simulation to clarify relevant assumptions for the understanding of joint models and assessment of its robustness under key model misspecifications. Further, we characterize the extent of bias introduced by the limiting assumptions of the time-varying covariate Cox model and compare its performance with a joint model in various contexts. We then present results of the two approaches to evaluate the potential of PTX3 as a prognostic marker of GvHD after haematopoietic stem cell transplantation.

**Results:**

Overall, we illustrate that a joint model provides an unbiased estimate of the association between a longitudinal marker and the hazard of an event in the presence of measurement error, showing improvement over the time-varying Cox model. However, a joint model is severely biased when the baseline hazard or the shape of the longitudinal trajectories are misspecified. Both the Cox model and the joint model correctly specified indicated PTX3 as a potential prognostic marker of GvHD, with the joint model providing a higher hazard ratio estimate.

**Conclusions:**

Joint models are beneficial to investigate the capability of the longitudinal marker to characterize time-to-event endpoint. However, the benefits are strictly linked to the correct specification of the longitudinal marker trajectory and the baseline hazard function, indicating a careful consideration of assumptions to avoid biased estimates.

## Background

The recent progress in molecular biology and genetics generates an increasing interest in investigating genomic or molecular biomarkers, as markers of diagnosis, prognosis or response to treatment. The longitudinal measure of biomarkers is useful for characterizing the occurrence of an outcome of interest, as they can be predictive of treatment results or related to the event process and prognosis. For example, the present work is motivated by a study, where the objective was to explore the potential of the long pentraxin 3 (PTX3) as a prognostic marker of Acute Graft-*versus*-Host Disease (GvHD) after haematopoietic stem cell transplantation [1].

The time-varying covariate Cox model (TVCM) [2, 3] has been used to study the association between an observed longitudinal measure of biomarkers and the hazard of an event [1, 4]. This approach uses the last-observation-carried-forward (LOCF), since marker’s observations are only available at discrete times (i.e. time of measurement), leading to the pitfall of introducing bias given the continuous nature of the biomarker [5]. Further, the TVCM fails to account for the so called “measurement error” in the biomarker. As evidenced by various studies (e.g., [6, 7]), failure to adjust for such measurement error introduces further bias into model estimates.

Shared frailty joint models address these issues by modeling simultaneously the profile of the marker and the time-to-event data [8, 9]. Within such approaches, a linear mixed model for the underlying longitudinal trajectories of the marker is linked to the survival model using shared random effects [10]. This approach allows inference on the association between the hazards of an event and the longitudinal biomarkers, by avoiding the LOCF assumption and accounting for the random measurement error [11]. However, joint models are parametric and thus require additional strong assumptions over the semi-parametric Cox model with time-varying covariate [12]. Assumptions are needed on both the distribution of the marker and its trajectory, and on the shape of the hazard function of the event of interest.

The literature that evaluates the impacts of misspecification of joint models for their applications in biomedical research has been particularly rare, while methodological efforts rapidly increasing (e.g., [13]). This causes a lack of clarity on practical issues, which in turn discourages applied researchers to improve the understanding of such models [14, 15]. Few simulation studies have been performed in the joint modeling framework. [16] investigated the use of joint models to adjust for measurement error only at the baseline measurement value. Simulation by [11] evaluated the performance of the joint model and the TVCM focusing on treatment effect on the time-to-event outcome, while [17] focused on the association between marker and event under few specific scenarios. A broader simulation study that evaluates the impact of model misspecifications and that could be useful for applied statisticians in order to understand advantages and disadvantages of a joint model as compared with a Cox model in different contexts is lacking. Moreover, the distinctive role of the bias due to LOCF and measurement error in the TVCM has not received attention in the previous studies. In this paper, we conduct a comprehensive simulation study with the following goals: (a) disentangle the bias introduced by LOCF and measurement error when assessing the association between a marker and a time-to-event endpoint by the TVCM and to compare its performance with a joint model, (b) clarify relevant assumptions of the joint model and assess its robustness in the presence of key model misspecifications, in particular considering the misspecifications of the marker distribution, of the marker trajectory, and of the shape of the hazard function. Additionally, these theoretical considerations will be used to evaluate the potential of PTX3 as a prognostic marker of GvHD after haematopoietic stem cell transplantation.

In “Method” section below, we describe the TVCM and the joint model approaches. In “Simulation study” section we present the simulation studies: simulation protocol, key model misspecification scenarios and discussion of associated results. In “Motivating context” section, we present an application to illustrate the use of PTX3 as a marker of GvHD using both the TVCM and joint model. Concluding discussion is presented in “Discussion” section.

## Method

### Notation

Let $T^{*}_{i}$ be the failure time of subject *i* (*i*=1,…,*n*) in a cohort of size *n*. Suppose that we want to estimate the association between a biomarker *w*_*i*_(*t*), that is varying in time, and the hazard of failure. In practice, the longitudinal biomarker is measured at discrete times *t*_*ij*_,*j*=1,…,*n*_*i*_. Thus, biomarker information coming from the *i*-th subject is a vector of observed discrete values, possibly subjected to the measurement error *ε*_*i*_(*t*), {*y*_*i*_(*t*_*ij*_)=*w*_*i*_(*t*_*ij*_)+*ε*_*i*_(*t*_*ij*_),*j*=1,…,*n*_*i*_}. Since survival times are commonly affected by right censoring, the observed survival time is $T_{i}=\text {min}(T^{*}_{i}, C_{i})$, where *C*_*i*_ is the right censoring time and $\delta _{i}=I(T^{*}_{i}\leq C_{i})$ is the event indicator, indicating whether the survival time or the censoring time is observed. $T^{*}_{i}$ and *C*_*i*_ are assumed to be independent conditional on the biomarker trajectory *w*_*i*_(*t*), as commonly done in survival analysis (e.g., [18]).

### The time-varying covariate Cox model

The TVCM is a generalization of the Cox model [2] accounting for covariates that can change value during the observation time. The proportional hazards model has the form

1 $$ h_{i}(t)=h_{0}(t)\exp\{\alpha y_{i}(t)\}   $$

where *h*_0_(*t*) denotes an unspecified baseline hazard, *α* is a parameter measuring the association between the observed longitudinal measure *y*_*i*_(*t*) and the hazard at time *t* (*h*_*i*_(*t*)). A vector of fixed baseline covariates can also be included in the model (1). The hazard ratio HR= exp(*α*) is interpreted as the relative increase in the hazard at any time *t* for a unit increase in the observed value of the biomarker at the same time point. The HR is assumed to be constant in time, thus we assume that the relative increase in the hazard for each unit increase in the biomarker is the same for all the observation time. Inference is based on maximizing the partial likelihood [3]. Of note, when *y*_*i*_(*t*) is not observed at time *t*, the most updated value is used: *y*_*i*_(*t*_*ij*_),*t*_*ij*_≤*t*<*t*_*ij*+1_, using the LOCF principle [8].

### Joint models

A joint model of longitudinal and survival data comprises two linked submodels: the longitudinal and the survival submodels [10, 19]. The longitudinal submodel specifies the trajectory of a biomarker over time. This is typically achieved using a linear mixed effects model [20] of the form:

2 $$ y_{i}(t)=w_{i}(t) + \epsilon_{i}(t)=\boldsymbol{\beta}^{T}\boldsymbol{f}_{i}(t) + \boldsymbol{b}^{T}_{i}\boldsymbol{g}_{i}(t)+ \epsilon_{i}(t)   $$

in which ***f***_*i*_(*t*) and ***g***_*i*_(*t*) are vectors of functions of time *t* for the fixed effect parameters ***β*** and the random effect parameters ***b***_*i*_, respectively. The component *ε*_*i*_(*t*) denotes mutually independent normally distributed error terms with variance $\sigma ^{2}_{\epsilon }$. For the random effects, one assumes ***b***_*i*_∼MVN(***0***,*Σ*), where *Σ* is inter-subject variance-covariance matrix. Further, the random effects are assumed to be independent of the error terms. In model (2) the observed marker value *y*_*i*_(*t*) at time point *t* is decomposed into the underlying true marker value *w*_*i*_(*t*) and a random error term. The survival submodel attempts to associate the marker value with the hazard of an event at the same time point *t* using the proportional hazards model:

3 $$ h_{i}(t)=h_{0}(t)\exp\{\alpha w_{i}(t)\}   $$

Similarly to (1), the parameter *α* measures the association between the longitudinal biomarker and the time-to-event and the hazard ratio HR= exp(*α*) is assumed constant in time. A vector of fixed baseline covariates can be included in this model as well. The basic difference with (1) is that model (3) does not use the observed value of the biomarker *y*_*i*_(*t*), but an estimate of the true value *w*_*i*_(*t*), which is continuously updated in time and obtained by maximizing the joint likelihood of the time-to-event and longitudinal marker outcomes. As a note, an appropriate estimate of the subject trajectory *w*_*i*_(*t*) requires correct specification of the design vectors ***f***_*i*_(*t*) and ***g***_*i*_(*t*). The optimization procedure involves a hybrid of expectation maximization (EM) and direct maximization as discussed in [10]. Unlike in the TVCM of (1), the baseline hazard must be specified parametrically or approximated by spline-based approaches. In fact, leaving the baseline hazard completely unspecified within the joint modeling framework severely underestimates the standard errors of the parameter estimates [21]. While the association parameter in both (3) and (1) is denoted by *α*, the corresponding estimates from the two models would be different.

## Simulation study

In this section, we conduct a simulation study under various scenarios in order to address the two aims, (a) disentangling the bias introduced by LOCF and measurement error when assessing the association between a marker and a time-to-event by the TVCM and compare its performance with that of the joint model. The second aim (b) focuses on clarifying relevant assumptions of the joint model and assess its robustness in the presence of model misspecifications. In fact, in the joint modeling framework, the association between the longitudinal marker and the hazard of an event relies on several assumptions on the longitudinal and survival submodels, including the marker distribution, the marker trajectory and the shape of the hazard function. The impacts of misspecifying these assumptions are illustrated, respectively, in the sections **b1**, **b2** and **b3**. Table 1 summarizes the main parameter values used for the simulation scenarios, which are described below. All simulations and analyses were performed using the R package JM version 1.4.7.

**Table 1:** Summary of the simulation protocol comprising main parameter values, marker and survival time distributions used for each of the simulation scenarios

Scenario	*β*_0_	*β*_1_	*β*_2_	***b***_*i*_	*Σ*_11_	*Σ*_22_	*Σ*_33_	*h*_0_(*t*)
a) LOCF and measurement error impact								
1	3.2	0	0	*N*(0,*Σ*_11_)	0	0	0	Weibull (0.1,1.4)
2	3.2	−0.07	0	*N*_2_(***0***,*Σ*)	1.44	0.04	0	Weibull (0.1,1.4)
b1) Marker distribution								
3	3.2	−0.07	0	BM ^∗^	1.44	0	0	Weibull (0.1,1.4)
4	3.2	−0.07	0	*χ*^2^(0.72)	1.44	0	0	Weibull (0.1,1.4)
5	3.2	−0.07	0	*Γ*(0.5,1.7)	1.44	0	0	Weibull (0.1,1.4)
6	3.2	−0.07	0	*N*(0,*Σ*_11_)	1.44	0	0	Weibull (0.1,1.4)
b2) Marker profile								
7	3.2	−0.07	0.004	*N*_3_(***0***,*Σ*)	1.44	0.6	0.09	Weibull (0.1,1.4)
8	3.2	−0.16	0.01	*N*_3_(***0***,*Σ*)	1.44	0.6	0.09	Weibull (0.1,1.4)
b3) Baseline hazard								
9	3.2	−0.07	0	*N*_2_(***0***,1.44)	1.44	0.04	0	*h*_0_(*t*)^*nonm*^

BM ^∗^ denotes a bimodal mixture distribution 0.65∗*N*(8,1.44)+0.35∗*N*(15,1.44)

*h*_0_(*t*)^*nonm*^=*νκt*^*κ*−1^/(*c*+*t*^*κ*^), where *ν*=1,*κ*=2,*c*=10

### Simulations protocol

We considered a sample size of *n*=300 subjects with regular measures of the biomarker for 14 weeks, including the baseline measurement (*t*=0,...14). The simulation setting was inspired by the motivating context of the data in “Motivating context” section. Data were generated by the following steps:

1. The general formula to obtain the true marker value *w*_*i*_(*t*) was given as
  4 $$ \begin{aligned} w_{i}(t)&=\beta_{0}+\beta_{1}t+\beta_{2}t^{2}+b_{i0}+b_{i1}t+b_{i2}t^{2} \\ &\boldsymbol{b}_{i}=(b_{i0},b_{i1}, b_{i2})^{T}\sim N_{3}(\boldsymbol{0}, \Sigma), \\ \end{aligned}   $$
  where *Σ* denotes 3 by 3 inter-subject variance-covariance matrix. When a linear decreasing trajectory was considered, as for the majority of the scenarios reported in Table 1, the fixed effect parameters were chosen to be *β*_0_=3.2, *β*_1_=−0.07 and *β*_2_=0. A basic scenario of biomarker with constant value in time was also considered by setting *β*_1_=*β*_2_=0 (scenario 1, Table 1). To assess the misspecification of the marker distribution (**b1**), a random intercept model was considered with *b*_*i*0_ generated from four different probability distributions: a Bimodal mixture of two normal distributions (hereafter called Bimodal), Chisquare, Gamma and Normal (scenarios 3 to 6). The parameter values of these distributions were chosen such that their corresponding variances equaled the random intercept variance *Σ*_11_=1.44. Model (4) was used to investigate misspecification of marker trajectory (**b2**) by generating biomarker values with a quadratic profile in scenarios 7 and 8, as depicted in Fig. 2a.

**Fig. 1:**
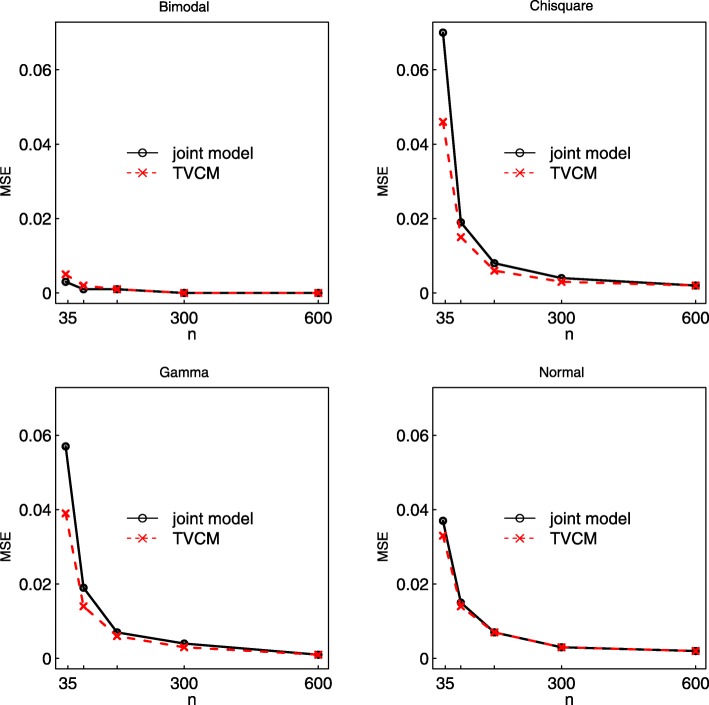
Mean-squared error (MSE) of the association parameter *α* obtained from the joint model and TVCM to the data generated considering different sample sizes (*n*) and different probability distributions for the random effect *b*_*i*0_

**Fig. 2:**
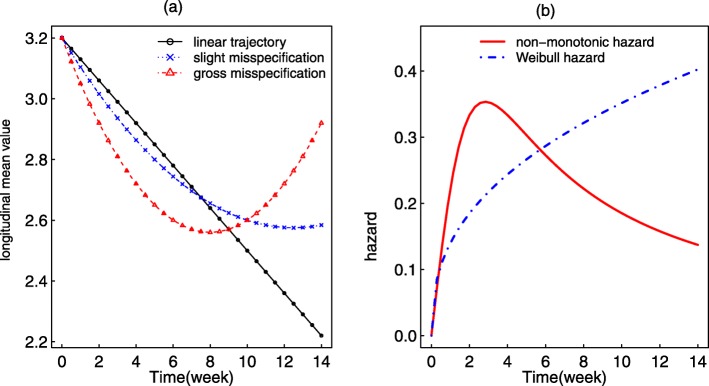
**a** Mean biomarker trajectory for the different scenarios: linearly decreasing (scenarios 2-6 and 9) and quadratic shape with slight (scenario 7) and gross (scenario 8) misspecifications with respect to the linear trend. **b** Baseline hazard function for the scenarios 1-8 (Weibull) and 9 (non-monotonic shape)
2. The observed marker value *y*_*i*_(*t*) at time *t* was obtained as *y*_*i*_(*t*)=*w*_*i*_(*t*)+*ε*, where *ε* represents a normally distributed measurement error $\epsilon \sim N(0,\sigma ^{2}_{\epsilon })$, with increasing variability *σ*_*ε*_∈(0.1,0.3,0.5), corresponding to a coefficient of variation (CV), defined as the standard deviation of measurement error divided by the mean (e.g.,[22]), of 3.1*%*,9.4*%*,15.6*%* respectively. Regular measures of *w*_*i*_(*t*) were obtained with increasing frequency, from one measurement per week (*t*=0,1,…,14) to 4 measurements per week (*t*=0,0.25,…,14), in order to examine the effect of LOCF in TVCM.
3. The survival time $T^{*}_{i}$ was obtained by a Weibull proportional hazard model: *h*_*i*_(*t*)=*λρt*^*ρ*−1^ exp{*αw*_*i*_(*t*)}, where *ρ*=1.4, *λ*=0.1. The association parameter was set at *α*∈(0,0.3,0.6), corresponding to no, moderate and strong association between *w*_*i*_(*t*) and *h*_*i*_(*t*), respectively. The survival time was generated by evaluating the inverse of a cumulative hazard (see, [23]). Since this does not lead to a closed form expression, we used the R root finder function uniroot to generate $T^{*}_{i}$ numerically. To investigate the impact of misspecifying the distribution of the hazard function on the association parameter *α* (**b3**), in the scenario 9, the survival times were generated from a non-monotonic baseline hazard function *h*_0_(*t*)=*νκt*^*κ*−1^/(*c*+*t*^*κ*^), where *ν*=1, *κ*=2 and *c*=10. The shape of this function, along with the Weibull curve previously described, were shown in Fig. 2b.
4. The censoring time *C*_*i*_ was generated according to a uniform distribution in (0,14), leading to around 20% of censoring proportion before week 14.
5. The observed survival time $T_{i}=min(T^{*}_{i},C_{i})$ was then calculated.
6. Marker values *y*_*i*_(*t*) with *t*>*T*_*i*_ were disregarded.

We drew *B*=1000 simulations for each scenario, *B* was chosen in order to get at least a 2% level of accuracy in the estimate of the association parameter *α* in about 95% of the samples, assuming a true association parameter of 0.6 with standard error 0.14 [24]. To each generated dataset, we fitted the following models: i) basic Cox model considering only the baseline measurement of a marker, *y*_*i*_(*t*=0); ii) the TVCM considering the observed updated value of the marker; iii) the joint model considering the updated value of the marker. We summarized the results using: the mean of the simulation estimates (Est), empirical Monte Carlo standard error (ESE), asymptotic standard error (ASE), percentage bias (%Bias =bias/*α*) and 95% coverage probabilities (CP) of the association parameter *α*. We also used bias and the mean-squared error (MSE) as necessary. The ASE was computed as the average of the estimated standard errors, and the ESE as the standard deviation of the estimates of *α*.

### Results

#### a) Measurement error and last observation carried forward impact

Table 2 shows the results of the constant biomarker case (scenario 1 of Table 1). The TVCM and the baseline Cox model show a very similar performance, with increasing bias as the measurement error is increasing. This is expected given that the biomarker mean value does not change over time. In the presence of small measurement error (*σ*_*ε*_=0.1), the joint model estimate showed a higher bias, indicating that a joint model is less beneficial in the presence of small measurement error and a constant biomarker. However, when *σ*_*ε*_ was increased to 0.3 and 0.5, the bias in the estimates of the joint model was smaller than the one in the TVCM, suggesting the ability of the joint model to account for measurement error.

**Table 2:** Results on the association parameter *α* obtained from the baseline Cox model, the TVCM and the joint model fitted to data generated considering a constant biomarker (scenario 1 of Table 1), *α*∈(0,0.3,0.6) and *σ*_*ε*_∈(0.1,0.3,0.5) with CV ∈(3.1*%*,9.4*%*,15.6*%*). Mean of the maximum likelihood estimates (Est), empirical Monte Carlo standard error (ESE), asymptotic standard error (ASE), percentage bias (%Bias) and 95% coverage probabilities (CP) are shown

		*α*=0.3				*α*=0.6					
*σ*_*ε*_	Model	Est	ESE	ASE	%Bias	CP	Est	ESE	ASE	%Bias	CP
	Cox.baseline	0.299	0.056	0.056	-0.3	95	0.598	0.060	0.060	-0.3	94
0.1	TVCM(1x/week)	0.299	0.056	0.056	-0.3	95	0.598	0.060	0.060	-0.3	94
	joint model	0.302	0.055	0.056	0.7	95	0.605	0.059	0.059	0.8	94
	Cox.baseline	0.283	0.054	0.055	-5.7	94	0.557	0.059	0.058	-7.2	87
0.3	TVCM(1x/week)	0.282	0.054	0.055	-6.0	94	0.557	0.057	0.058	-7.2	88
	joint model	0.302	0.057	0.057	0.7	95	0.606	0.062	0.061	1.0	95
	Cox.baseline	0.254	0.052	0.052	-15	85	0.489	0.056	0.054	-18	45
0.5	TVCM(1x/week)	0.252	0.051	0.052	-16	84	0.489	0.053	0.054	-18	46
	joint model	0.302	0.059	0.058	0.7	95	0.607	0.068	0.066	1.2	94

Table 3 shows the results under scenario 2 (linearly decreasing marker), with *α*∈(0,0.3,0.6). The ESE (not reported) were always in close agreement with the ASE. When *α* was set at 0, a similar good performance of the three models was visible regardless of the size of *σ*_*ε*_. In the other scenarios, we can observe increasing bias, and decreasing coverage probabilities, for the TVCM (every week) as the magnitude of *σ*_*ε*_ increases. With *σ*_*ε*_=0.1 and *α*=0.3, the percentage bias was −2.3*%* and the coverage 95%. This percentage bias raised to −19*%*, and the coverage dropped to 80%, when *σ*_*ε*_ increased to 0.5, while it reduced to −0.7*%* when the number of measurements taken was increased to four times per week, thus the impact of LOCF estimate was reduced. The advantage of using the joint model was observed in the presence of high measurement error, where the percentage bias of −19*%* (TVCM) was reduced to 0.3*%*. The joint model, fitted using the parametric Weibull baseline hazard, provided the most unbiased estimates with coverage probabilities much closer to 95% across all the scenarios. We note that the performance of the TVCM falls further in the presence of a strong association between the marker and the time-to-event. For instance, with *α*=0.6 and *σ*_*ε*_=0.5, a large percentage bias, −21*%*, and a very small coverage, 35%, were observed for the TVCM (once per week). In the latter setting, the improvement achieved by increasing the number of measurements was small.

**Table 3:** Results of the association parameter *α* obtained from the baseline Cox model, the TVCM and the joint model fitted to data generated considering the linear marker trajectory (scenario 2 of Table 1) with *α*∈(0,0.3,0.6) and *σ*_*ε*_∈(0.1,0.3,0.5) with CV ∈(3.1*%*,9.4*%*,15.6*%*). Mean of the maximum likelihood estimates (Est), asymptotic standard error (ASE), bias, percentage bias (%Bias) and 95% coverage probabilities (CP) are shown

		*α*=0				*α*=0.3				*α*=0.6			
*σ*_*ε*_	Model	Est	ASE	Bias	CP	Est	ASE	%Bias	CP	Est	ASE	%Bias	CP
	Cox.baseline	0.000	0.060	0.000	95	0.254	0.056	-15	86	0.545	0.059	-9.2	83
0.1	TVCM(1x/week)	-0.003	0.060	-0.003	96	0.293	0.058	-2.3	95	0.582	0.061	-3.0	93
	TVCM(4x/week)	-0.001	0.060	-0.001	95	0.298	0.059	-0.7	94	0.594	0.062	-1.0	94
	joint model	-0.003	0.059	-0.003	96	0.301	0.058	0.3	95	0.604	0.061	0.7	94
	Cox.baseline	0.000	0.058	0.000	96	0.240	0.054	-20	79	0.508	0.057	-15	62
0.3	TVCM(1x/week)	-0.003	0.058	-0.003	96	0.275	0.057	-8.3	92	0.541	0.059	-9.8	83
	TVCM(4x/week)	-0.001	0.058	-0.001	95	0.279	0.057	-7.0	92	0.550	0.059	-8.3	86
	joint model	-0.003	0.060	-0.003	95	0.301	0.059	0.3	95	0.604	0.064	0.7	94
	Cox.baseline	0.000	0.055	0.000	96	0.216	0.051	-28	61	0.449	0.053	-25	23
0.5	TVCM(1x/week)	-0.003	0.055	-0.003	96	0.244	0.053	-19	80	0.474	0.055	-21	35
	TVCM(4x/week)	-0.002	0.055	-0.002	95	0.247	0.054	-18	84	0.480	0.055	-20	40
	joint model	-0.004	0.062	-0.004	95	0.301	0.062	0.3	95	0.606	0.069	1.0	94

#### b) Results under model misspecification

##### b1) Marker distribution

In joint modeling, the marker distribution is typically assumed to be Gaussian (e.g., [16]). Violation of this assumption is a key concern as the random effects play a central role in characterizing the association between the biomarker and hazard of an event [10]. The simulation study in this section assesses the effect of misspecifying the distribution of the random effects according to the scenarios 3 to 6 of Table 1. A random intercept model was considered to generate the random intercept *b*_*i*0_ from three non-normal distributions and a reference Normal distribution. The joint model was fitted assuming a normally distributed random intercept in the longitudinal submodel. Five different sample sizes of 35, 75, 150, 300 and 600 subjects were considered in this setting. The measurement error standard deviation was held fixed *σ*_*ε*_=0.3 and the true association parameter *α*=0.3. The results of the simulation are shown in Table 4. The joint model failed to converge for a few simulations with small sample size: 6/1000 when the data were generated using the Bimodal distribution with *n*=35 and 1/1000 for *n*=75. These non-converging simulations were excluded from the analyses. When the marker was generated from a non-normal distribution, the joint model produced a biased estimate of *α* for *n*=35, with a percentage bias of 22%, 17% and 7.7*%* when the random intercept was generated from Chisquare, Gamma and Bimodal distributions, respectively. However, the percentage bias decreased as the sample size *n* increased, reaching a maximum value of 3.7*%* with *n*=600 subjects, and the coverage probabilities were closer to the optimal 95% across all distributions. Further, both the ESE and the ASE decreased as the sample size increased. Thus, the estimate of the association between longitudinal marker and hazard of an event is not affected substantially by the misspecification of the random effect distribution as long as the sample size is large.

**Table 4:** Results of the association parameter *α* obtained from joint model and TVCM fitted to data generated considering the sample size *n*∈(35,75,150,300,600) and different probability distributions (scenarios 3:6 of Table 1) for the random effect *b*_*i*0_ with variance *Σ*_11_=1.44, *α*=0.3 and *σ*_*ε*_=0.3 with CV =9.4*%*

		Joint model					TVCM(1x/week)				
Distribution	*n*	Est	ESE	ASE	%Bias	CP	Est	ESE	ASE	%Bias	CP
	35	0.315	0.191	0.179	5.0	95	0.287	0.182	0.176	-4.3	95
	75	0.308	0.123	0.116	2.7	94	0.286	0.117	0.114	-4.7	95
Normal	150	0.306	0.085	0.081	2.0	94	0.285	0.081	0.079	-5.0	94
	300	0.302	0.057	0.056	0.7	95	0.282	0.055	0.055	-6.0	94
	600	0.304	0.040	0.040	1.3	95	0.284	0.038	0.039	-5.3	93
	35	0.323	0.054	0.051	7.7	95	0.315	0.066	0.062	5.0	96
	75	0.309	0.033	0.033	3.0	95	0.303	0.038	0.038	1.0	95
Bimodal	150	0.305	0.023	0.023	1.7	95	0.301	0.025	0.025	0.3	96
	300	0.302	0.016	0.016	0.7	96	0.299	0.017	0.018	-0.3	96
	600	0.302	0.011	0.011	0.7	96	0.299	0.012	0.012	-0.3	95
	35	0.366	0.256	0.211	22	93	0.309	0.215	0.195	3.0	95
	75	0.334	0.134	0.125	11	94	0.297	0.121	0.118	-1.0	96
Chisquare	150	0.316	0.088	0.083	5.3	94	0.287	0.079	0.079	-4.3	95
	300	0.318	0.059	0.057	6.0	94	0.289	0.054	0.054	-3.7	95
	600	0.309	0.040	0.039	3.0	95	0.283	0.036	0.037	-5.7	94
	35	0.352	0.232	0.200	17	93	0.305	0.197	0.187	1.7	96
	75	0.327	0.135	0.122	9.0	93	0.291	0.120	0.116	-3.0	96
Gamma	150	0.316	0.079	0.081	5.3	96	0.287	0.073	0.077	-4.3	96
	300	0.316	0.061	0.056	5.3	94	0.289	0.056	0.054	-3.7	94
	600	0.311	0.041	0.039	3.7	94	0.286	0.037	0.037	-4.7	95

Mean of the maximum likelihood estimates (Est), empirical Monte Carlo standard error (ESE), asymptotic standard error (ASE), percentage bias (%Bias) and 95% coverage probabilities (CP) are shown

The TVCM is relatively less biased and more precise in the estimate of *α* for small sample sizes, indicating it could provide a good accuracy even though the marker was contaminated with a measurement error (*σ*_*ε*_=0.3). Figure 1 shows the MSE for the joint and TVCM models under the four distributions. The MSE reflects the accuracy of each model taking into account both the bias and variability [24]. For the small sample size, the TVCM has lower MSE, except for the Normal case where MSE from both models are the same. As the sample size increases, the MSE from both models coincide.

##### b2) Marker trajectory

In order to appropriately characterize the association between the marker and the hazard of an event, estimation of the subject-specific trajectory *w*_*i*_(*t*) from (2) must capture the underlying shape. To evaluate the impact of misspecification of the marker profile on the estimate of *α*, we generated longitudinal trajectories which were quadratic in nature and fitted a joint model assuming linear trajectories with random intercept and random slope. We considered a slight and a gross departure from linearity, with parameters specified in scenarios 7 and 8 of Table 1, respectively. Figure 2a illustrates the mean longitudinal profile under both scenarios.

Table 5 reports the results of the simulation study under marker trajectory misspecification. The table includes the TVCM fitted to the generated observed longitudinal marker based on four times per week. A lack of convergence was encountered for the joint model under gross misspecification: the frequencies of non-convergence were 16/1000 and 13/1000 for *σ*_*ε*_=0.3 and *σ*_*ε*_=0.5, respectively. Further, one extreme outlier estimate for each of the two *σ*_*ε*_ values was obtained. The two outliers were excluded from the results shown in Table 5. The impact of the marker trajectory misspecification is clearly observed in the estimates of the joint model. For *σ*_*ε*_=0.3, we observe a percentage bias of −5.3*%* for the joint model under slight misspecification. This corresponds to an extra 5% bias as compared with the same scenario when the marker shape was correctly specified (see, Table 3). The extra bias could be as large as 8.7*%* under gross misspecification. These indicate that the longitudinal trajectory of a marker must be carefully specified when a joint model is considered for estimating the association between longitudinal biomarker and time-to-event. In the event of gross misspecification, the TVCM provides less biased estimates even in the presence of moderate measurement error in the biomarker.

**Table 5:** Results of the association parameter *α* estimated from the TVCM and joint model fitted to data generated considering slight and gross misspecifications of the longitudinal trajectories (scenarios 7 and 8 of Table 1), *σ*_*ε*_∈(0.1,0.3,0.5) with CV ∈(3.1*%*,9.4*%*,15.6*%*) and the true *α*=0.3

		Slight misspecification					Gross misspecification				
*σ*_*ε*_	Model	Est	ESE	ASE	%Bias	CP	Est	ESE	ASE	%Bias	CP
0.1	TVCM(1x/week)	0.295	0.056	0.056	-1.7	95	0.297	0.055	0.056	-1.0	95
	TVCM(4x/week)	0.309	0.054	0.055	3.0	96	0.310	0.053	0.054	3.3	96
	joint model	0.297	0.209	0.060	-1.0	92	0.280	0.222	0.059	-6.7	91
0.3	TVCM(1x/week)	0.277	0.054	0.054	-7.7	93	0.280	0.053	0.054	-6.7	93
	TVCM(4x/week)	0.292	0.051	0.053	-2.7	95	0.294	0.051	0.052	-2.0	95
	joint model	0.284	0.291	0.067	-5.3	91	0.273*	0.290	0.059	-9.0	91
0.5	TVCM(1x/week)	0.249	0.050	0.051	-17	83	0.252	0.050	0.051	-16	84
	TVCM(4x/week)	0.264	0.047	0.050	-12	89	0.266	0.047	0.049	-11	90
	joint model	0.265	0.192	0.071	-12	91	0.265*	0.167	0.065	-12	91

Mean of the maximum likelihood estimates (Est), empirical Monte Carlo standard error (ESE), asymptotic standard error (ASE), percentage bias (%Bias) and 95% coverage probabilities (CP) are shown. The star (*) indicates that one extreme outlier estimate was removed

##### b3) Hazard shape function

Within the joint model framework, leaving the baseline hazard unspecified severely underestimates the standard errors of the parameter estimates [21]. Thus the hazard function for the survival submodel is often assumed to be Weibull (e.g., [25]), but the evolution of the hazard rate over time can easily be non-monotonic (e.g., [26, 27]). To investigate the impact of misspecifying the distribution of the hazard function on the association parameter *α*, we generated data following a non-monotonic hazard (scenario 9 in Table 1) and fitted the joint model assuming three baseline hazard shapes: constant, Weibull and splines. For the case of splines, the baseline hazard was defined using B-splines (e.g., [28]) with 5 internal knots placed at equally-spaced percentiles of the observed survival time *T*_*i*_. Table 6 reports the results considering *α*∈(0.3,0.6) and *σ*_*ε*_∈(0.1,0.3,0.5). The performance of the TVCM was comparable to the previous scenarios (see Table 3), while the accuracy of the joint model was strictly dependent on the assumptions on the hazard shape. The joint model with constant hazard produced severely biased estimates: for example when *σ*_*ε*_=0.1, *α*=0.3 was underestimated by 39%, with a coverage of 39%, and none of the confidence intervals contained the true value, when *α* was set at 0.6. Thus, even if the constant hazard can be appealing for ease of computation, it often does not represent a realistic assumption. When the joint model was fitted to the generated data assuming a Weibull hazard, the estimate of *α* was also biased for all the scenarios. For *α*=0.3 and *σ*_*ε*_=0.1, *α* was overestimated by 12%. Joint models based on spline functions provided the most unbiased estimates of *α* with coverage probability closer to 95% in most scenarios. The flexibility of spline functions allowed to capture the underlying non-linear shape of the baseline hazard.

**Table 6:** Results of the association parameter *α* obtained from joint model and TVCM fitted to data generated considering a non-monotonic baseline hazard function (scenario 9 of Table 1), *α*∈(0.3,0.6) and *σ*_*ε*_∈(0.1,0.3,0.5) with CV ∈(3.1*%*,9.4*%*,15.6*%*)

		*α*=0.3					*α*=0.6				
*σ*_*ε*_	Model	Est	ESE	ASE	%Bias	CP	Est	ESE	ASE	%Bias	CP
0.1	TVCM(1x/week)	0.292	0.058	0.058	-2.7	94	0.579	0.062	0.061	-3.5	93
	joint-constant	0.183	0.041	0.054	-39	39	0.336	0.035	0.052	-44	0
	joint-weibull	0.337	0.066	0.058	12	86	0.638	0.065	0.061	6.3	89
	joint-spline	0.303	0.060	0.059	1.0	95	0.608	0.064	0.063	1.3	94
0.3	TVCM(1x/week)	0.274	0.056	0.056	-8.7	92	0.538	0.059	0.058	-10.3	80
	joint-constant	0.180	0.043	0.055	-40	39	0.328	0.037	0.053	-45	0
	joint-weibull	0.340	0.068	0.059	13	87	0.642	0.070	0.065	7.0	89
	joint-spline	0.304	0.062	0.061	1.3	95	0.608	0.077	0.067	1.3	94
0.5	TVCM(1x/week)	0.244	0.053	0.053	-18	81	0.471	0.055	0.055	-21	36
	joint-constant	0.174	0.046	0.052	-42	39	0.312	0.04	0.055	-48	0
	joint-weibull	0.344	0.071	0.062	15	85	0.643	0.164	0.071	7.2	89
	joint-spline	0.304	0.065	0.064	1.3	95	0.615	0.197	0.073	2.5	94

Mean of the maximum likelihood estimates (Est), empirical Monte Carlo standard error (ESE), asymptotic standard error (ASE), percentage bias (%Bias) and 95% coverage probabilities (CP) are shown

## Motivating context

The example is coming from a study where patients with haemato-oncological diseases who underwent stem cell transplantation (HSCT) were evaluated to explore the potential of the long pentraxin 3 (PTX3) as a prognostic marker of Acute Graft-*versus*-Host Disease (GvHD) [1]. Acute graft-versus-host disease is one of the major causes of morbidity and mortality associated with allogeneic stem cell transplants [29]. Currently, the diagnosis of GvHD is based on clinical signs and symptoms and requires invasive biopsies of disease target organs in uncertain cases, which are sometimes unfeasible. To improve diagnosis and prognosis of GvHD, recent researches focus on specific biomarkers measured in the plasma or serum of HSCT patients as a new tool for detecting GvHD prior to clinical manifestation and for GvHD management. PTX3 is an acute-phase protein, rapidly produced by vascular endothelial cells, mesenchymal cells and fibroblasts, as well as by innate immune response cells upon stimulation with pro-inflammatory cytokines, damaged tissue-derived signals and microbial antigens. Differently from other acute phase proteins, such as the C-Reactive Protein, PTX3 is considered a rapid marker for primary local activation of innate immunity and inflammation due to its peculiar pattern of production.

In this section, we compare the use of the TVCM and joint model for the evaluation of PTX3 as a marker of GvHD. Peripheral blood samples were collected in a cohort of 116 patients before the beginning of conditioning regimen, on day 0 (HSCT), weekly after HSCT until the 14*th* week and at the development of symptoms consistent with GvHD. Plasma was obtained after centrifuging whole blood and PTX3 was evaluated by Sandwich ELISA assay, with a measurement precision declared as an intra-assay CV lower than 10%. The median follow-up time was 5 weeks. Time was measured from HSCT up to the occurrence of GvHD, censoring occurred if a subject died before GvHD or was lost to follow-up. The follow-up ended at the 14*th* week.

**Fig. 3:**
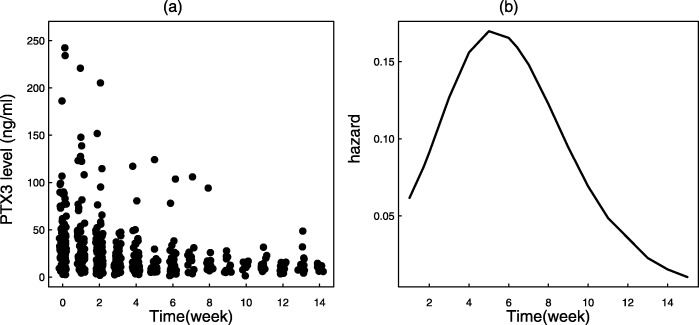
**a** The distribution of PTX3 marker in time. **b** The shape of the distribution of the GvHD hazard estimate

Figure 3a displays the distribution of the PTX3 marker over time, showing a decreasing trend and departure of the distribution from normality. The average PTX3 at week 0 for all subjects was 29.46 *ng*/*ml* (nanograms per milliliter) with a standard deviation of 31.5. The GvHD hazard was estimated using the bshazard package [30], and plotted in Fig. 3b, which showed a highly non-monotonic shape of the GvHD event. We fitted a TVCM and a joint model to evaluate the association between the marker and the hazard of GvHD. Consistently with the simulation study, we also considered the basic Cox model that uses only the baseline information, observed at *t*=0, as a covariate. For the joint model the longitudinal PTX3 was specified using a linear mixed model with random intercept and random slope, which was chosen as the best model according to AIC selection criterion when compared to a mixed model that involves a quadratic time. The baseline hazard within the joint model was specified as constant, Weibull and B-splines with 6 internal knots placed at equally-spaced percentiles of the event time. Each model was fitted considering both the original PTX3 and the logarithmic transformation of PTX3 to satisfy the normality assumption of the linear mixed model.

The results are shown in Table 7, which reports the estimated association between PTX3 and GvHD (Est), the standard error of the estimate (SE), hazard ratio (HR), and the 95% confidence interval of the HR (95% HR CI). The baseline marker did not showed a significant association with the hazard of GvHD event. The updated values of PTX3 appear to be positively associated with the hazard of the GvHD as estimated by the TVCM, with both its original value and the log transformed version, even though the HR values are not comparable due to the log transformation. The TVCM hazard ratio of 1.14 indicates that a unit increase in the PTX3 marker corresponds to a 1.14-fold increase in the hazard of developing the GvHD disease.

**Table 7:** Estimates of the association of PTX3, and log(PTX3), with time to GvHD from the baseline Cox model, TVCM and joint model

	PTX3				log(PTX3)			
Model	Est	SE	HR	95% HR CI	Est	SE	HR	95% HR CI
Cox.baseline	-0.08	0.04	0.92	(0.85,1.00)	-0.17	0.14	0.84	(0.64, 1.11)
TVCM	0.13	0.03	1.14	(1.08,1.20)	0.60	0.14	1.82	(1.38, 2.41)
joint-constant	0.05	0.08	1.05	(0.95,1.21)	-0.28	0.29	0.76	(0.42, 1.36)
joint-Weibull	0.11	0.01	1.11	(0.84,1.47)	0.79	0.49	2.20	(0.84,5.78)
joint-spline	0.13	0.12	1.14	(0.90,1.44)	1.13	0.55	3.11	(1.05, 9.18)

The estimated association between PTX3 and GvHD (Est), the standard error of the estimate (SE), the hazard ratio (HR), and the 95% confidence interval of the HR (95% HR CI) are reported

The joint models using constant and Weibull hazards estimated a lower non-significant association between PTX3 and time to GvHD. Interestingly, when the hazard was modeled by splines, the HR point estimate was equal to the one obtained by the TVCM (1.14), but with higher variability. When the log of PTX3 was used in a joint model with spline baseline hazard, a HR(95%CI) of 3.11(1.05,9.18) was obtained. It follows that a unit increase in the log of PTX3 marker was associated to a 3.11-fold increase in the risk of developing the GvHD disease. This value was greater than the HR of 1.82 estimated by the TVCM, but with higher variability.

Overall, we notice a great variability among the joint model estimates of the HR, ranging from 0.76 up to 3.11. This can be directly linked to the misspecification of the marker and hazard distribution in some of the applied models, coherent with the simulation results. The Cox model was unaffected by the normality of the marker and from the hazard distribution.

**Fig. 4:**
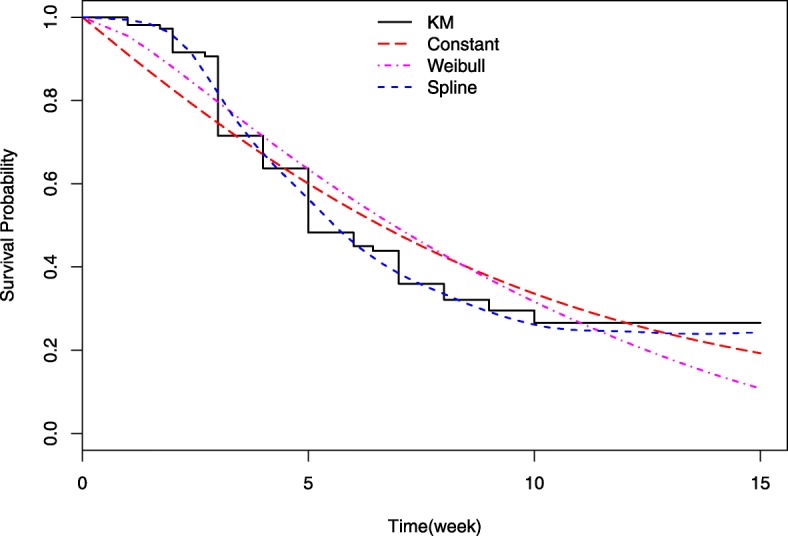
Observed Kaplan-Meier (KM) curve and predicted survival curves from the joint model assuming constant, Weibull and spline based hazards. A logarithmic transformation of PTX3 was used in the joint models

Figure 4 shows the Kaplan-Meier (KM) estimate of GvHD occurrence and the predicted marginal survival from each one of the applied joint models. The spline based survival curve was much closer to the KM curve, suggesting that splines were able to capture the strong non-linear hazard function shown in Fig. 3b. The curve associated to the Weibull was in agreement with the KM estimate until the 4*th* week of follow-up, but the difference with the KM estimate increased over time. As expected, the survival curve associated to the constant hazard largely deviated from the KM curve.

## Discussion

Investigating biological biomarkers as markers of diagnosis/prognosis or response to treatment requires inferential tools for the association between the longitudinal process of the marker and the progression of the diseases. The TVCM has been the standard approach, but its partial likelihood assumes constant biomarker values between follow-up times and ignores measurement error. There has been some effort to expand the Cox model to accommodate measurement error, such as regression calibration (e.g., [33]), that however requires the availability of a validation subsample, that is not often available. The modeling of the longitudinal profile of the biomarker by a linear mixed model is another approach to obtain an estimate of the expected value of the biomarker free from measurement error, that can be included as a covariate in the TVCM with a two-stage approach [17]. Joint models simultaneously analyze the longitudinal marker profile and the time to an event overcoming both the issues of LOCF and measurement error. Joint models are, however, computationally intensive and require additional assumptions over the TVCM. In this paper, we performed a comprehensive simulation study with the goal of clarifying relevant assumptions for the understanding of a joint model and for assessing its robustness under key model misspecifications. Further, we disentangled the bias introduced by LOCF and measurement error in the TVCM and compared its performance with the joint model. Overall, we illustrated that the TVCM approach underestimates the association estimates in the presence of measurement error.The major source of the TVCM bias was attributable to measurement error compared with that attributable to LOCF. On the other hand, the joint model can be severely biased under model misspecification.

Firstly we considered how estimates from a joint model may be biased under the misspecification of the normality assumption for the true marker distribution. Violation of this assumption for joint models is an issue as the random effects play a central role for characterizing the association between the marker and the hazard of an event [10]. To avoid parametric distributional assumption, joint models based on a semi-parametric [31] or non-parametric assumptions [5] have been proposed. Further, [32] showed that parameter estimates are robust to misspecification as the number of measurements per subject increases. We showed that the misspecification has a negligible effect on the estimate of the association parameter as long as the sample size is large, regardless of the parametric distribution being adopted. The TVCM was not affected by the marker distribution. This is expected, but it is worth to underline it here to discourage unnecessary log-transformation to account for normality in the Cox model framework, which is sometimes seen in the medical literature (e.g., [34]).

Second, we looked into the impact of misspecifying the longitudinal marker trajectory on the estimate of the association between the marker and the hazard of an event. This is motivated by the fact that the true underlying marker trajectory is typically unknown, since we only observe error contaminated and intermittently measured marker. To effectively characterize the association estimate, the true marker trajectory must be appropriately estimated [10]. We illustrated that failing to capture the underlying marker trajectory, at different amounts of measurement error, leads to substantially biased estimates in the joint model, while the TVCM is unaffected by the misspecification, since it does not assume any form of marker shape. [17] similarly found that, at fixed measurement error, estimates from the joint model are biased under marker trajectory misspecification. However, they also suggested that the bias is still less than the bias from the TVCM.

Furthermore, we found that a misspecification of the baseline hazard in the joint modeling framework has an important effect on the estimate of the association between the longitudinal marker and the hazard of an event. This issue had never been considered in the literature of joint models, but simulations indicated that the association estimate was severely biased when the data generating hazard process was misspecified. This was particularly evident when we attempted to model a highly non-linear hazard shape by a constant or Weibull hazard. On the other hand, association estimate using TVCM was insensitive to the misspecification of the baseline hazard, as its shape is unspecified. In the joint modeling framework leaving the baseline hazard unspecified severely underestimates the standard error of the parameters [21], even if it appears to be the most applied choice as shown in a recent meta-analysis on joint models [25]. Thus, the baseline hazard in the joint model should be carefully modeled, also with the use of splines if necessary, to avoid bias on the association estimate. The two modeling techniques were illustrated using a real data on HSCT for establishing PTX3 as a marker of GvHD. The joint model, with the hazard modeled by spline functions, provided the PTX3 as a potential diagnostic marker of GvHD. This was corroborated by the TVCM, even if it indicated a lower association estimate.

In conclusion, joint models are a powerful tool, able to account for marker measurement error and to model the marker trajectory in time. However, they require strong assumptions that need to be properly validated, and the avoidance of bias due to model misspecification is crucial in order for a joint model to provide a substantive benefit over the semi-parametric Cox model with a time-varying covariate. Furthermore, it may be suggested that the better performance by the joint model is unfair because the data generating scheme in our simulation utilized a biomarker measurement error whereas the TVCM does not assume the presence of measurement error. We showed that the performance of the joint model was higher than that of a TVCM accounting for measurement error in the biomarker by a two-stage approach, while requiring similar hypotheses. The results are provided in the Additional file 1.

## Supplementary information

**Additional file 1** Supplementary Appendix.

## Data Availability

The datasets along with the simulation code used during the current study are available from the corresponding author on reasonable request.

## Abbreviations

ASE: Asymptotic standard error
CI: Confidence interval
CP: Coverage probabilities
ESE: Empirical monte carlo standard error
Est: Mean of the maximum likelihood estimates
GvHD: Acute graft-versus-host disease
HR: Hazard ratio
HSCT: Haemato-oncological stem cell transplantation
KM: Kaplan-meier
LOCF: Last observation carried forward
PTX3: Long Pentraxin 3
TVCM: Time-varying covariate cox model
