# Supplementary material for: Joint model robustness compared with the time-varying covariate Cox model to evaluate the association between a longitudinal marker and a time-to-event endpoint
Source: BMC Med Res Methodol. 2019 Dec 3;19:222. doi: 10.1186/s12874-019-0873-y (PMC6888912; doi:10.1186/s12874-019-0873-y)
Supplement: Supplementary file 1 — Additional file 1 Supplementary Appendix. [file 12874_2019_873_MOESM1_ESM.pdf]

Joint model robustness compared with the time-varying covariate Cox model to evaluate the association between a longitudinal marker and a time-to-event endpoint

Maeregu W. Arisido, Laura Antolini, Davide P. Bernasconi, Maria G. Valsecchi and Paola Rebora

Supplementary Appendix

Results of the association parameter α obtained from the time-varying covariate Cox model (TVCM), two-stage approach and the joint model fitted to data generated considering the linear marker trajectory (scenario 2 of Table 1 in the manuscript with $\alpha \in (0, 0.3, 0.6)$ and $\sigma_\epsilon \in (0.1, 0.3, 0.5)$. Mean of the maximum likelihood estimates (Est), asymptotic standard error (ASE), bias, percentage bias (%Bias) and 95% coverage probabilities (CP) are shown.

		$\alpha = 0.3$				$\alpha = 0.6$			
σ_ϵ	Model	Est	ASE	%Bias	CP	Est	ASE	%Bias	CP
	TVCM	0.293	0.058	-2.3	95	0.582	0.061	-3.0	93
0.1	Two-stage	0.285	0.059	-5	94	0.575	0.062	-4.2	92
	joint model	0.301	0.058	0.3	95	0.604	0.061	0.7	94
	TVCM	0.275	0.057	-8.3	92	0.541	0.059	-9.8	83
0.3	Two-stage	0.280	0.06	-6.7	93	0.567	0.063	-5.5	92
	joint model	0.301	0.059	0.3	95	0.604	0.064	0.7	94
	TVCM	0.244	0.053	-19	80	0.474	0.055	-21	35
0.5	Two-stage	0.273	0.062	-9	92	0.547	0.065	-8.8	86
	joint model	0.301	0.062	0.3	95	0.606	0.069	1.0	94
